# The COVID‐19 pandemic impact on wellbeing and mental health in people with psychotic and bipolar disorders

**DOI:** 10.1002/brb3.2559

**Published:** 2022-04-06

**Authors:** Elizabeth Ann Barrett, Carmen Simonsen, Sofie Ragnhild Aminoff, Wenche Ten Velden Hegelstad, Trine Vik Lagerberg, Ingrid Melle, Erlend Mork, Kristin Lie Romm

**Affiliations:** ^1^ Early Intervention in Psychosis Advisory Unit for South East Norway (TIPS Sør‐Øst) Division of Mental Health and Addiction Oslo University Hospital Oslo Norway; ^2^ Norwegian Centre for Mental Disorders Research (NORMENT) Institute of Clinical Medicine University of Oslo Oslo Norway; ^3^ TIPS Centre for Clinical Research in Psychosis Stavanger University Hospital Stavanger Norway; ^4^ Institute of Social Studies Faculty of Social Sciences University of Stavanger Stavanger Norway; ^5^ Norwegian Centre for Mental Disorders Research (NORMENT) Division of Mental Health and Addiction Oslo University Hospital Oslo Norway

**Keywords:** bipolar disorder, COVID‐19, mental health, psychotic disorders, schizophrenia

## Abstract

**Introduction:**

The COVID‐19 pandemic affects people globally, but it may affect people with psychotic and bipolar disorders disproportionally. Our aims were to investigate the pandemic impact on perceived wellbeing and mental health in this population, including which pandemic‐related factors have had an impact.

**Methods:**

People with psychotic and bipolar disorders (*N* = 520; female = 81%; psychotic disorders *n* = 75/bipolar disorder *n* = 445) completed an online survey about wellbeing and mental health in the early phase of the COVID‐19 pandemic (June 5–July 5, 2020).

**Results:**

Many participants experienced deteriorated wellbeing and mental health after the pandemic outbreak, especially in life satisfaction, meaning in life, positive feelings, depression, anxiety, and self‐harm/suicidal ideation. Experienced recovery from mental health difficulties was significantly lower after compared to before the outbreak. Participants with psychotic disorders had significantly poorer wellbeing and mental health than participants with bipolar disorders, although they experienced significantly more worsening only of psychotic symptoms. Nearly half the participants reported coping with the situation; however, most factors potentially important to wellbeing and mental health changed adversely, including sufficiency and quality of treatment. More loneliness, low coping, insufficient mental health treatment during the COVID‐19 pandemic, pandemic worry, more insomnia symptoms, and increased alcohol use predicted poor wellbeing and poor mental health.

**Conclusions:**

During a pandemic, it is particularly important that mental health services strive to offer the best possible treatment under the current conditions and target loneliness, coping strategies, pandemic worry, insomnia, and increased alcohol use to uphold wellbeing and reduce mental health difficulties. For some, teletherapy is an agreeable substitute for traditional therapy.

## Graphical abstract

The majority of people with psychotic and bipolar disorders experienced deteriorated wellbeing and mental health during the early phase of the COVID‐19 pandemic. Experienced recovery from mental health difficulties was significantly lower after compared to before the outbreak. Poor wellbeing and poor mental health was associated with pandemic‐related changes, including more loneliness, low coping with the situation, insufficient mental health treatment, pandemic worry, more insomnia symptoms, and increased alcohol use.

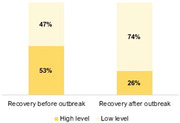

## 1 INTRODUCTION

The Coronavirus disease‐19 (COVID‐19) developed fast into a pandemic with increasing death rates worldwide. Most governments have initiated public measures to prevent the spread of COVID‐19, including wearing face masks, physical/social distancing, and self‐isolation (WHO, 2020b). The pandemic has an unprecedented impact on people's lives by causing worry about contracting the virus and challenges living with social precautions. This may have negative consequences for peoples’ mental health (Holmes et al., 2020; Kumar & Nayar, 2021). Indeed, studies report that distress, disturbed sleep, anxiety, and depression have been common reactions in the general population during the current and previous virus outbreaks (e.g., the Severe Acute Respiratory Syndrome [SARS] outbreak in 2002–2004 and the Middle East Respiratory Syndrome [MERS] outbreak in 2012) (Alkhamees et al., 2020; Rajkumar, 2020b; Salari et al., 2020; Torales et al., 2020; Vindegaard & Benros, 2020). The pandemic implications for people's wellbeing, that is, positive feelings, life satisfaction, meaning in life, and social connectedness (Chan et al., 2018) are more uncertain. Alongside potential negative effects, people may experience a renewed sense of shared purpose in the joint combat of the virus (Brown et al., 2020), and feelings of happiness, freedom, and an increased sense of calm have been reported resulting from a slower pace of life (Simblett et al., 2021).

Although the pandemic affects people globally, there are concerns that it impacts people with severe mental disorders disproportionately. Severe mental disorders include psychotic disorders (PsD; characterized by presence of psychotic symptoms) and bipolar disorders (BD; characterized by the presence of (hypo)manic and depressive symptoms). Psychotic disorders and bipolar disorders overlap in terms of symptomatology, with around 50% having secondary affective symptoms and psychotic symptoms, respectively (Romm et al., 2010; Simonsen et al., 2011). Thus, they can be regarded dimensionally rather than categorically different. People with PsD and BD may be more vulnerable during the pandemic due to higher sensitivity to stress, smaller social networks, high prevalence of substance use, sensitivity to circadian rhythm disruption, and dependency of social, community, and mental health services, which are all factors potentially affected by the pandemic (Brown et al., 2020; Holmes et al., 2020; Kozloff et al., 2020; Rajkumar, 2020a). Distress, loneliness, sleep problems, change in substance use, practical/financial problems, and lack of treatment may reduce wellbeing and affect mental health by exacerbation of anxiety, depression, suicidality, mania, or psychosis. Based on PsD and BD overlapping in terms of symptomatology, it is of interest to investigate whether or how they potentially differ in response to the life‐changing situation brought about by the pandemic.

Some studies have investigated the impact of the COVID‐19 outbreak on this population, with reports of higher levels of distress, alcohol use, sleep disruption, anxiety, depression, and poorer coping strategies compared to healthy controls (González‐Blanco et al., 2020; Solé et al., 2020; Van Rheenen et al., 2020), with 30% showing symptom relapse and 5% reporting increased suicidality after the outbreak (Muruganandam et al., 2020). However, Pinkham et al. (2020) found no change in affective or psychotic symptoms and an increase in wellbeing in people with schizophrenia spectrum and affective disorders. Thus, the findings are somewhat inconsistent, with little knowledge about pandemic impact on wellbeing and psychotic symptoms specifically. Increased knowledge about pandemic effects may suggest measures that could mitigate negative consequences during the ongoing and future pandemics.

The primary aims of this study were thus to investigate the COVID‐19 impact on the experience of both wellbeing and mental health difficulties in people with PsD and BD, including which pandemic‐related factors have had an impact.

## 2 MATERIALS AND METHODS

### 2.1 Setting and procedures

The Norwegian Government announced a nationwide lockdown on March 12, 2020 in order to reduce the spread of COVID‐19. Protecting healthcare professionals was highlighted, with the intention to uphold healthcare services (Government.no, 2020a). Physical distancing (2 m indoors/1 m in public spaces) and good hand hygiene were emphasized, and number of people allowed to meet was restricted. The general recommendation was to “avoid contact with other people.” People who tested positive for COVID‐19 were to be isolated, and those who had been exposed to the virus had to quarantine. Day‐care centers, educational institutions, fitness centers, swimming pools, and establishments providing hair and body care were closed. Restaurants, bars, etc. were closed except where visitors could keep a distance of at least 1 m. Cultural events, sporting events, organized sporting activities, and visits to holiday properties (e.g., summerhouses) were prohibited. Use of public transportation and all non‐essential travels were advised against. Healthcare workers were prohibited from traveling abroad. Access to public healthcare facilities was restricted, and visits were prohibited. One‐to‐one health services (e.g., private mental health therapy, physiotherapy, etc.) that could not uphold physical distancing were closed. On March 17, 2020, the Government urged healthcare services to implement the use of digital tools, including videoconference (Government.no, 2020b). Following the Government's announcement, general practitioners advised against attendance unless strictly necessary. Mental health services canceled services, except those considered necessary to avoid severe exacerbation and life‐threatening behavior. However, in line with the Government's recommendation, mental healthcare services gradually acquired and increased the use of digital tools/teletherapy. In sum, everyday life was highly affected by the lockdown, but Norway's pandemic control was regarded as efficient, with comparatively low infection and death rates (Sachs et al., 2020). From April 7, 2020, the precautions were gradually lifted, including reopening of day‐care centers and schools, reopening one‐to‐one health services in accordance with guidelines for infection control standards, reducing the social distancing rule from 2 to 1 m, increasing the number of people allowed to gather, etc. For an overview and timeline of restrictions, see: https://eurohealthobservatory.who.int/monitors/hsrm/all‐updates/hsrm/norway/physical‐distancing

The authors and people with lived experience of mental health difficulties created an online survey in “Nettskjema” (University of Oslo, 2020). The people with lived experience provided input on content and phrasing of questions, as well as piloting the survey. All their input was taken into account. The survey was distributed from Oslo University Hospital (OUS) and Stavanger University Hospital via Facebook and Instagram, as well as via clinician networks, user organizations, and charities. We published the survey June 5–July 5, 2020, when society was still gradually reopening. The survey included an initial section informing about its purpose and that pressing “Next page” indicated consent to participate. Responses were anonymous. The study was approved by the Regional Committees for Medical and Health Research Ethics (reference number 140012), and by the Data Protection Officer at OUS (reference number 20/12120).

### 2.2 Participants

People with PsD and BD were invited by OUS to respond to how they experienced the COVID‐19 pandemic. The first item in the survey was to check off for the diagnostic group that the participants identified with. We chose to use the term “psychotic disorders” rather than the diagnostic equivalents (e.g., F20–F29 in the ICD‐10 [WHO, 1992]), because this is a more commonly used term in Norway. There were no other inclusion or exclusion criteria.

### 2.3 Measurements

In the survey, participants were informed that the questions concerned the situation during the COVID‐19 pandemic, meaning after March 12, 2020. Questions about demographics covered age, gender, place of birth, immigration background, education, and marital status.

Wellbeing was measured using a Norwegian guideline list for wellbeing measurement (Nes et al., 2018), which is based on the OECD Guidelines on Measuring Subjective Well‐being (OECD, 2013) and Diener's Flourishing Scale (Diener et al., 2009). Questions about wellbeing covered life satisfaction, meaning in life, social support, and positive feelings. To assess mental health, we used the items from the Patient Health Questionnaire‐4 (PHQ‐4) (Kroenke et al., 2009) to measure anxiety symptoms (anxiousness, worry) and depressive symptoms (low mood, little interest/pleasure). All other questions were made for this survey. The additional questions concerning mental health covered symptoms of self‐harm/suicidal ideation, mania, and psychosis. Participants rated all questions about wellbeing and mental health at the time of the survey (“now” or “past 2 weeks”) on a scale from 0 (not at all) to 10 (to a great extent), thus the original PHQ‐4 scale from 0 to 3 was changed to 0–10, to be in line with the other outcome scales. Additionally, the participants rated whether each wellbeing and mental health item had changed after the pandemic outbreak. Participants also rated their experience of recovery (i.e., improvement) from mental health difficulties (1) before and (2) after the outbreak on a 0 (not at all) −10 (to a large extent) scale.

Questions about factors potentially important to wellbeing and mental health and affected by the pandemic covered worry about pandemic consequences, coping with the pandemic situation, keeping updated about the pandemic situation via different channels, and adherence to government recommendations. Moreover, the survey included questions about change from before to after the pandemic outbreak in housing situation, daily activity, personal economy, social life, social isolation, and family conflicts. Participants rated feelings of loneliness the past 2 weeks on a 0 (not at all)–10 (to a large extent) scale, and also whether feelings of loneliness had changed after the outbreak. The survey included questions about change in substance use and medication use from before to after the outbreak. It also included questions about insomnia symptoms and troubling nightmares the past 2 weeks and change since before the outbreak, as well as bedtime and rise‐time before and after the outbreak. Regarding treatment for psychotic and bipolar disorders, the survey included questions about mental health service providers, community and charity mental health support, and mode of treatment delivery before and after the outbreak, and also whether participants would like to continue with new modes of treatment delivery. Furthermore, we asked whether participants had received sufficient treatment during the pandemic and about the quality of treatment after compared to before the outbreak. Finally, the survey asked whether participants had refrained from contacting mental health services and whether they had called helplines after the outbreak. An English translation of the survey questions and response alternatives (except PHQ‐4 items and demographics) relevant for this paper are presented in Appendix A.

### 2.4 Statistical analysis

Statistical analyses were performed with IBM SPSS Statistics 26. Analyses were two‐tailed with a pre‐set significance level of .05. Diagnostic group differences were analyzed with *t*‐test, Mann–Whitney *U*‐test, chi‐square test, and Fischer's exact test according to analyses assumptions. Changes since before the outbreak (i.e., differences between pre and post outbreak ratings, *or* ratings of items having become “worse/no change/better”) were analyzed with McNemar's test, Wilcoxon Signed Rank test, or chi‐square test. In order to reduce the chance of type 1 errors, we ran a series of Bonferroni corrections on bivariate comparisons of PsD and BD, and bivariate comparisons of pandemic‐related factors before and after the pandemic. Thus, we divided the pre‐set significance level of .05 with the number of tests run for a certain area. Pandemic‐related predictors of “Poor wellbeing” (no/yes) and “Poor mental health” (no/yes) were investigated with two series of binary logistic regression analyses. Poor wellbeing was assigned if either one of the following items had a score ≤5 *and* being “worse” post outbreak: Life satisfaction, meaning in life, social support, or being happy. Poor mental health was assigned if either one of the following items had a score ≥5 *and* being worse post outbreak: anxious, worried, depressed mood, little interest/pleasure, self‐harm/suicidal ideation, ideas of persecution, or hallucinations. The independent variables were factors potentially important for wellbeing and mental health that had changed during the pandemic and differed significantly between the no/yes poor wellbeing and no/yes poor mental health participants in bivariate analyses. To avoid multicollinearity, only the most relevant variable from each domain was selected. Controlling for potential confounders, diagnosis, age, gender, education, and being single were included in the analyses. Some variables were dichotomized for the regression analyses: *Gender* (“Female” vs. “Not” [male/other]); *Marital status* (“Single” [single, divorced/separated, widowed] vs. “Partner” [girlfriend/boyfriend, married/cohabitant]); and *More alcohol use* (“No” [no use, less use, a lot less use] vs. “Yes” [more use, a lot more use]).

To facilitate interpretation some variables were also reversed: treatment sufficiency = *Insufficient treatment* (“No” vs. “Yes/Uncertain”); coping with the situation = *Low coping* (“No” [not at all/a little] vs. “Yes” [a lot]; and Personal economy = *Poorer economy* (“No” [a lot better, somewhat better, no change] vs. “Yes” [a lot worse, somewhat worse]). Relevant independent variables were entered into the regression analyses for each of the two dependent variables, taking out those that did not have a significant contribution one by one.

## 3 RESULTS

### 3.1 Demographics

Five hundred and twenty participants completed the survey (BD, *n* = 445; PsD, *n* = 75). Demographic data are presented in Table 1. The only significant diagnostic group differences were BD participants having higher education and being married or cohabitant more often than the PsD participants.

**TABLE 1:** Demographic data for the total sample and differences across diagnostic groups

	Total sample, *N* = 520	Bipolar disorders, *n* = 445	Psychotic disorders, *n* = 75	Test statistics^a^	*p*
Age, M (SD)	36.8 (12.3)	37.1 (12.0)	35.0 (14.0)	*t* = 1.4	.176
Gender, *n* (%)					
‐Female	420 (81)	358 (80)	62 (83)		
‐Male	95 (18)	84 (19)	11 (15)		
‐Other	5 (1)	3 (1)	2 (3)		.165
Place of birth, *n* (%)					
‐Norway	485 (93)	416 (94)	69 (92)	*χ* ^2^ = 0.1	.635
‐Abroad	35 (7)	29 (7)	6 (8)		
Immigration background,^b^ *n* (%)					
‐No	492 (95)	420 (94)	72 (96)		
‐Yes	28 (5)	25 (6)	3 (4)		.608
Education, *n* (%)					
‐Compulsory school (10 years)	49 (9)	**35** (**8**)	**14** (**19**)		
‐High school (13 years)	195 (38)	**157** (**35**)	**38** (**51**)		
‐1–2 years university	79 (15)	69 (16)	10 (13)		
‐Bachelor's degree	133 (26)	**124** (**28**)	**9** (**12**)		
‐Master's degree or higher	64 (12)	60 (14)	4 (5)	*χ* ^2^ = 22.0	**<.001**
Marital status *n* (%)					
‐Single	205 (39)	**156** (**35**)	**49** (**65**)		
‐Girlfriend/boyfriend	63 (12)	54 (12)	9 (12)		
‐Married/cohabitant	209 (40)	**197** (**44**)	**12** (**16**)		
‐Divorced/separated	41 (8)	37 (8)	4 (5)		
‐Widowed	2 (0.4)	1 (0.2)	1 (1)		**<.001**

*Note*: Bold numerals indicate statistically significant differences between participants with psychotic‐ and bipolar disorders.

*Abbreviations*: M, mean; SD, standard deviation.

^a^ Test statistics: *t*‐test, Mann–Whitney *U*‐test; chi‐square, Fischer's exact test.

^b^ Immigration background = both parents being born abroad.

### 3.2 Wellbeing and mental health difficulties

Data on wellbeing and mental health difficulties are presented in Table 2. The majority of participants experienced low levels of wellbeing; life satisfaction, meaning in life, and positive feelings, with many experiencing worsening post COVID‐19 outbreak. Almost half the participants reported low levels of social support, but few experienced worsening. PsD participants experienced significantly lower levels of life satisfaction, meaning in life, and feeling happy compared to the BD participants.

**TABLE 2:** Wellbeing and mental health difficulties after the COVID‐19 outbreak and change since before the outbreak^a^ in the total sample and across diagnostic groups

Wellbeing^b^					
‐Measured on a scale from 0 (“not at all”) to 10 (“to a large degree”)	Total sample *N* = 520	Bipolar disorder *n* = 445	Psychotic disorder *n* = 75	Test statistics^c^	*p*
Life satisfaction					
‐Low score ≤ 5, *n* (%)	331 (64)	272 (61)	59 (79)	*χ* ^2 ^= 7.8	**.005**
‐M (SD)	4.8 (2.6)	4.8 (2.5)	3.4 (2.8)	*t* = 4.4	**<.001**
‐Worse/no change/better, *n* (%)	234 (45)/206 (40)/80 (15)	192 (43)/179 (40)/74 (17)	42 (56)/27 (36)/6 (8)	*χ* ^2^ = 5.8	.056
Meaning in life					
‐Low score ≤ 5, *n* (%)	337 (65)	277 (62)	60 (80)	*χ* ^2 ^= 8.1	**.004**
‐M (SD)	4.4 (2.7)	4.52 (2.7)	3.4 (2.8)	*t* = 3.4	**.001**
‐Worse/no change/better *n* (%)	188 (36)/269 (52)/63 (12)	159 (36)/229 (51)/57 (13)	29 (39)/40 (53)/6 (8)	*χ* ^2^ = 1.4	.491
Social support					
‐Low score ≤ 5, *n* (%)	226 (44)	185 (42)	41 (55)	*χ* ^2 ^= 4.0	*.047*
‐M (SD)	5.8 (2.8)	5.91 (2.7)	5.33 (3.0)	*t* = 1.7	.094
‐Worse/no change/better, *n* (%)	89 (17)/354 (68)/77 (15)	71 (16)/305 (68)/69 (16)	18 (24)/49 (65)/8 (11)	*χ* ^2^ = 3.5	.170
Positive feelings^4^					
**Happy**					
‐Low score ≤ 5, *n* (%)	332 (64)	277 (62)	55 (73)	*χ* ^2 ^= 3.0	.065
‐M (SD)	4.6 (2.4)	4.7 (2.3)	4.0 (2.5)	*t* = 2.5	**.013**
**Engaged**					
‐Low score ≤ 5, *n* (%)	358 (69)	303 (68)	55 (73)	*χ* ^2 ^= 0.6	.440
‐M (SD)	4.4 (2.5)	4.4 (2.5)	3.8 (2.7)	*t* = 1.9	.057
**Calm and relaxed**					
‐Low score ≤ 5, *n* (%)	388 (75)	330 (74)	58 (77)	*χ* ^2 ^= 0.2	.659
‐M (SD)	3.8 (2.6)	3.9 ((2.5)	3.6 (2.9)	*t* = 0.8	.407
Positive feelings					
‐Worse/no change/better, *n* (%)	259 (50)/200 (38)/61 (12)	218 (49)/173 (39)/54(12)	41 (55)/27 (36)/7 (9)	*χ* ^2^ = 1.0	.612

Mental health difficulties^d^					
Measured on a scale from 0 (“not at all”) to 10 (“to a large degree”)	Total sample *N* = 520	Bipolar disorder *n* = 445	Psychotic disorder *n* = 75	Test statistics^c^	*p*
Anxious^e^					
‐High score ≥5, *n* (%)	369 (71)	312 (70)	57 (76)	*χ* ^2^ = 0.8	.367
‐Mdn (min−max)	7 (0–10)	6 (0–10)	7 (0–10)	*U* = 19,088.0	*.045*
‐Worse/no change/better, *n* (%)	262 (50)/218 (42)/40 (8)	219 (49)/190 (43)/36 (8)	43 (57)/28 (37)/4 (5)	*χ* ^2^ = 1.9	.384
Worried^e^					
‐High score ≥5 *n* (%)	336 (65)	279 (63)	57 (76)	*χ* ^2^ = 4.4	*.036*
‐MDN (min−max)	6 (0–10)	5 (0–10)	7 (0–10)	*U* = 20,166.0	**.004**
‐Worse/no change/better, *n* (%)	226 (44)/260 (50)/34 (7)	185 (42)/229 (52)/31 (7)	41 (55)/31 (41)/3 (4)		.100
Depressed mood^e^					
‐High score ≥5 *n* (%)	361 (69)	305 (69)	56 (75)	*χ* ^2^ = 0.9	.352
‐M (SD)	6.0 (2.9)	5.9 (2.8)	6.4 (3.2)	*t* = −1.3	.195
‐Worse/no change/better, *n* (%)	254 (49)/212 (41)/54 (10)	212 (48)/185 (42)/48 (11)	42 (56)/27 (36)/6 (8)	*χ* ^2^ = 1.9	.389
Little interest/pleasure^e^					
‐High score ≥5, *n* (%)	306 (59)	265 (60)	41 (55)	*χ* ^2^ = 0.5	.504
‐M (SD)	5.0 (2.9)	5.0 (3)	5.1 (3.5)	*t* = −0.5	.627
‐Worse/no change/better %	200 (38)/278 (54)/42 (8)	174 (39)/235 (53)/36 (8)	26 (35)/43 (57)/6 (8)	*χ* ^2^ = 0.6	.750
Self‐harm/suicidal ideation					
‐High score ≥5, *n* (%)	211 (41)	170 (38)	41 (55)	*χ* ^2^ = 6.6	**.007**
‐Mdn (min−max)	3 (0–10)	3 (0–10)	5 (0–10)	*U* = 20,371.5	**.002**
‐Worse/no change/better, *n* (%)	173 (33)/312 (60)/35 (7)	138 (31)/276 (62)/31 (7)	35 (47)/36 (48)/4 (5)	*χ* ^2^ = 7.1	*.029*
Elevated mood					
‐High score ≥5, *n* (%)	156 (30)	140 (32)	16 (21)	*χ* ^2^ = 2.7	.102
‐Mdn (min−max)	2 (0–10)	2 (0–10)	1 (2–10)	*U* = 14,700.0	.089
‐More/no change/less, *n* (%)	105 (20)/284 (55)/131 (25)	93 (21)/240 (54)/112 (25)	12 (16)/44 (59)/19 (25)	*χ* ^2^ = 1.0	.598
Irritable mood					
‐High score ≥5, *n* (%)	292 (56)	254 (57)	38 (51)	*χ* ^2^ = 0.8	.363
‐M (SD)	4.8 (3.2)	4.9 (3.2)	4.4 (3.3)	*t* = 1.1	.275
‐More/no change/less, *n* (%)	259 (50)/221 (43)/40 (8)	224 (50)/183 (42)/38 (9)	35 (47)/38 (51)/2 (3)	*χ* ^2^ = 4.4	.109
Increased activity					
‐High score ≥5, *n* (%)	188 (36)	165 (37)	23 (31)	*χ* ^2 ^= 0.9	.348
‐Mdn (min−max)	3 (0–10)	3 (0–10)	2 (0–10)	*U* = 16,052.5	.590
‐More/no change/less *n* (%)	166 (32)/238 (46)/116 (22)	140 (32)/203 (46)/102 (23)	26 (35)/35 (47)/14 (19)	*χ* ^2 ^= 0.7	.690
Derealization					
‐High score ≥5, *n* (%)	114 (22)	81 (18)	33 (44)	*χ* ^2^ = 23.5	**<.001**
‐Mdn (min−max)	0 (0–10)	0 (0–10)	4 (0–10)	*U* = 22,471.5	**<.001**
‐Worse/no change/better, *n* (%)	88 (17)/426 (82)/6 (1)	**67** (**15**)/374 (**84**)/4 (1)	**21** (**28**)/52 (**69**)/2 (3)	*χ* ^2^ = 9.8	**.007**
Ideas of self‐reference					
‐High score ≥5, *n* (%)	39 (8)	25 (6)	14 (19)	*χ* ^2^ = 15.8	**<.001**
‐Mdn (min−max)	0 (0–10)	0 (0–10)	0 (0–10)	*U* = 20,710.5	**<.001**
‐Worse/no change/better, *n* (%)	30 (6)/486 (94)/4 (1)	21 (5)/421 (95)/3 (1)	9 (12)/65 (87)/1 (1)		*.037*
Ideas of persecution					
‐High score ≥5, *n* (%)	106 (20)	74 (17)	32 (43)	*χ* ^2^ = 25.2	**<.001**
‐Mdn (min−max)	0 (0–10)	0 (0–10)	3 (0–10)	*U* = 23,122.0	**<.001**
‐Worse/no change/better, *n* (%)	80 (15)/423 (81)/17 (3)	65 (15)/369 (83)/11 (3)	15 (20)/54 (72)/6 (8)		*.020*
Ideas of grandiosity					
‐High score ≥5, *n* (%)	56 (11)	37 (8)	19 (25)	*χ* ^2^ = 17.6	**<.001**
‐Mdn (min−max)	0 (0 – 10)	0 (0–10)	1 (0–10)	*U* = 22,172.0	**<.001**
‐Worse/no/better, *n* (%)	30 (6)/477 (92)/13 (3)	**20** (**5**)/415 (**93**)/10 (2)	**10** (**13**)/62 (**83**)/3 (4)		**.007**
Hallucinations					
‐High score ≥5, *n* (%)	73 (14)	42 (9)	31 (41)	*χ* ^2^ = 51.5	**<.001**
‐Mdn (min−max)	0 (0–10)	0 (0–10)	2 (0–10)	*U* = 24,214.0	**<.001**
‐Worse/no change/better, *n* (%)	47 (9)/460 (89)/13 (3)	**32** (**7**)/405 (**91**)/8 (**2**)	**15** (**20**)/55 (**73**)/5 (**7**)		**<.001**
Chaotic thinking					
‐High score ≥5, *n* (%)	361 (69)	307 (69)	54 (72)	*χ* ^2^ = 0.2	.698
‐Mdn (min−max)	7 (0–10)	7 (0–10)	8 (0–10)	*U* = 18,843.0	.071
‐Worse/no change/better, *n* (%)	227 (44)/266 (51)/27 (5)	189 (43)/232 (52)/24 (5)	38 (51)/34 (45)/3 (4)		.438

*Note*: Bold numerals indicate statistically significant difference between participants with psychotic‐ and bipolar disorders. Due to Bonferroni correction the significance levels were changed as follows: Wellbeing items (0.05/4) = 0.013; Depressive/anxiety symptoms (0.05/5) = 0.01; Psychotic symptoms (0.05/6) = 0.008. Italics numerals indicate no longer statistically different after Bonferroni correction.

*Abbreviations*: M, mean; Mdn, median; SD, standard deviation.

^a^ Before the COVID‐19 outbreak = before March 12, 2020; after the COVID‐19 outbreak = June 5–July 5, 2020.

^b^ Wellbeing was measured with the Norwegian guideline list for measurement of wellbeing.

^c^ Test statistics: *t*‐test, Mann–Whitney *U*‐test; chi‐square, Fischer's exact test.

^d^ Past 2 weeks.

^e^Patient Health Questionnaire‐4.

The majority of participants reported high levels of feeling anxious, worried, depressed, little interest/pleasure, irritable, and experiencing chaotic thinking, with around half of the participants reporting worsening post outbreak. PsD participants reported feeling significantly more worried and experienced more self‐harm/suicidal ideation, derealization, ideas of self‐reference, ideas of persecution, ideas of grandiosity, and hallucinations than BD participants. PsD participants also experienced more worsening of derealization, ideas of grandiosity and hallucinations.

**FIGURE 1:**
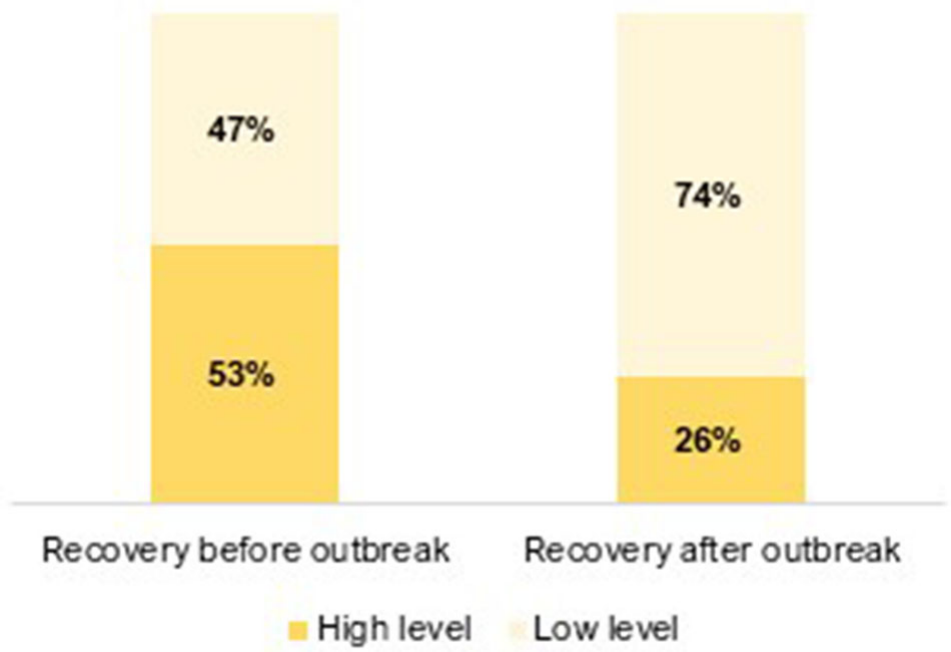
Participants experience of recovery^†^ (i.e., improvement) from their mental health difficulties before and after the COVID‐19 outbreak^‡^ (*N* = 520). ^†^Experience of recovery from mental health difficulties was measured on a 0 (“not at all”)–10 (“to a great extent”) scale; low level ≤ 5, high level ≥ 6. ^‡^Before the COVID‐19 outbreak = before March 12 2020; after the COVID‐19 outbreak = June 5–July 5, 2020. Change from before to after the COVID‐19 outbreak was analyzed with McNemars’ test (*χ* ^2^ = 81.5, *p* < .001). 53% (*n* = 275); 47% (*n* = 245) / 26% (*n* = 133); 74% (*n* = 387)

The participants’ experience of recovery (i.e., improvement) from mental health difficulties was significantly reduced from before to after the COVID‐19 outbreak (see Figure 1). Furthermore, the median recovery experience score was reduced from 6 to 3 (0–10 scale) from before to after the outbreak (*z* = −9.7, *p* < .001). PsD participants had lower median recovery scores post outbreak than BD participants (1 vs. 3; *U* = 13,793.0, *p* = .015).

### 3.3 Pandemic‐related factors

#### 3.3.1 Concerns and coping

**FIGURE 2:**
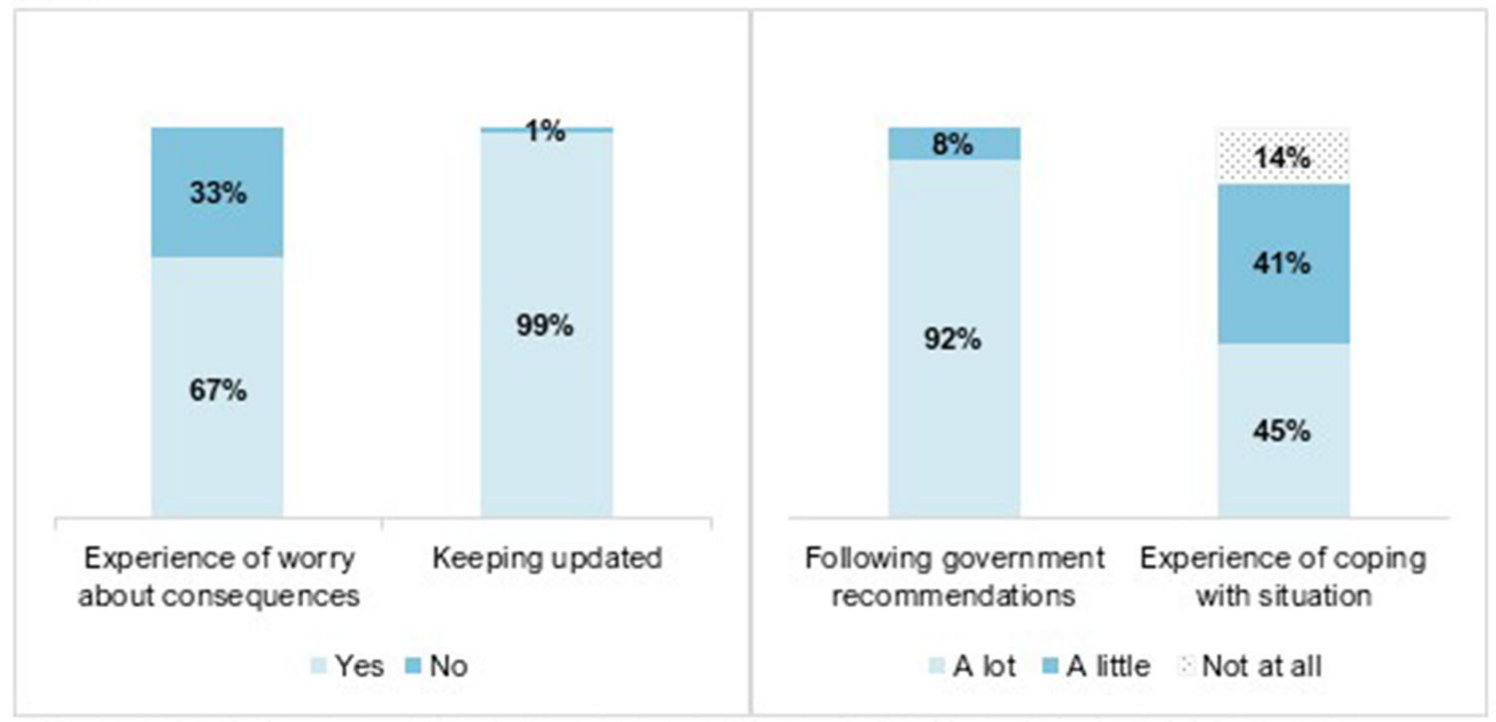
Participants experience of worry about pandemic consequences, keeping updated^†^, following Government recommendations^‡^, and coping with the pandemic situation during the COVID‐19 pandemic^§^ (*N* =  520). ^†^Keeping updated about the outbreak via either “health authorities webpages” (90%), “newspapers” (83%), “TV/radio” (78%), “social media” (83%), “blogs” (9%), or “friends/family” (84%). ^‡^Recommendations about hand washing and social distancing. ^§^During the COVID‐19 pandemic = between March 12, 2020 and June 5–July 5, 2020. 67% (*n* = 347); 33% (*n* = 173) / 99% (*n* = 514); 1% (*n* = 6) / 92% (*n* = 480); 8% (*n* = 39); 0.2% (*n* = 1) / 45% (*n* = 234); 41% (*n* = 216); 14% (*n* = 70)

Figure 2 shows that the majority of participants worried about COVID‐19 pandemic consequences, coped “a little” or “a lot,” kept updated, and followed Government recommendations. There was a numerical small but statistically significant group difference in keeping updated about the pandemic situation (PsD, *n* = 71 [95%] vs. BD, *n* = 445 [100%], *p* = .005).

#### 3.3.2 Housing, daily activity, and economy

Table 3 shows that in the BD group there was a significant increase in living with family/cohabitant from before to after the outbreak. There was also a significant reduction in studying and full‐time work and increase in temporarily lay‐offs and “other” activities. One‐in‐three reported worsened personal economy after the outbreak (see Table 4).

**TABLE 3:** Changes in housing and daily activity from before to after the COVID‐19 outbreak^a^ in the total sample and across diagnostic groups

	Total sample *N* = 520			Bipolar disorders *n* = 445			Psychotic disorders *n* = 75		
Change in housing from before to after the COVID‐19 outbreak, *n* (%)	Before	After	*p*	Before	After	*p*	Before	After	*p*
‐Alone	168 (32)	159 (31)	.151	134 (30)	126 (28)	.170	34 (45)	33 (44)	1.000
‐With family/cohabitant	305 (59)	322 (62)	.*012*	277 (62)	295 (66)	**.005**	28 (37)	27 (36)	1.000
‐With friends/shared accommodation	34 (7)	24 (5)	*.041*	31 (7)	20 (5)	*.019*	3 (4)	4 (5)	1.000
‐Supported housing	12 (2)	15 (3)	.375	2 (0.4)	4 (1)	.500	10 (13)	11 (15)	1.000
‐No residence	1 (0.2)	0	1.000	1 (0.2)	0 (0)	1.000	0 (0)	0 (0)	–

Change in daily activity from before to after the COVID‐19 outbreak, *n* (%)	Before	After	*p*	Before	After	*p*	Before	After	*p*
‐Studying	74 (14)	50 (10)	**<.001**	66 (15)	43 (10)	**<.001**	8 (11)	7 (9)	1.000
‐Part time work	82 (16)	73 (14)	.233	70 (16)	63 (14)	.337	12 (16)	10 (13)	.688
‐Full time work	115 (22)	95 (18)	**.001**	110 (25)	90 (20)	**.001**	5 (7)	5 (7)	1.000
‐Unemployed	63 (12)	60 (12)	.648	41 (9)	39 (9)	.815	22 (29)	21 (28)	1.000
‐Temporarily lay‐off	4 (1)	23 (4)	**<.001**	3 (1)	20 (5)	**<.001**	1 (1)	3 (4)	.500
‐Other	182 (35)	219 (42)	**<.001**	155 (35)	190 (43)	**<.001**	27 (36)	29 (39)	.688

*Note*: Bold numerals indicate statistically significant change from before to after the COVID‐19 outbreak. Italic numerals indicate no longer statistically different after Bonferroni correction. Due to Bonferroni correction, the significance level was changed: (.05/8) = .006. Changes from before to after the COVID‐19 outbreak were analyzed with McNemar's test.

^a^ Before the COVID‐19 outbreak = before March 12, 2020; after the COVID‐19 outbreak = June 5–July 5, 2020.

**TABLE 4:** Pandemic‐related factors: Changes in factors potentially important to wellbeing and mental health difficulties from before to after the COVID‐19 outbreak^a^ in the total sample and across diagnostic groups

	Total sample, *N* = 520	Bipolar disorders, *n* = 445	Psychotic disorders, *n* = 75	Test statistics^b^	*p*
Personal economy					
‐Worse/no change/better, *n* (%)	170 (33)/288 (55)/62 (12)	143 (32)/251 (56)/51 (12)	27 (36)/37 (49)/11 (15)	*χ* ^2 ^= 1.4	.490
Social life					
‐Worse/no change/better, *n* (%)	345 (66)/119 (23)/56(11)	300 (67)/98 (22)/47 (11)	45 (60)/21 (28)/9 (12)	*χ* ^2 ^= 1.7	.437
Social isolation^c^	*n* = 486	*n* = 415	*n* = 71		
‐More/no change/less, *n* (%)	370 (76)/84 (17)/32 (7)	322 (78)/68 (16)/25 (6)	48 (68)/16 (22)/7 (10)		.154
Feeling outside of the community^c^	*n* = 467	*n* = 397	*n* = 70		
‐More/no change/less, *n* (%)	321 (69)/103 (22)/43 (9)	269 (68)/92(23)/36 (9)	52 (74)/11 (16)/7 (10)	*χ* ^2 ^= 1.9	.382
Family conflict^c^	*n* = 396	*n* = 338	*n* = 58		
‐More/no change/less *n* (%)	149 (38)/196 (49)/51 (13)	130 (38)/162 (48)/46 (14)	19 (33)/34 (59)/5 (8)	*χ* ^2 ^= 2.5	.283
Loneliness^d^ (0–10)					
‐High score ≥ 6, *n* (%)	284 (55)	234 (53)	49 (65)	*χ* ^2 ^= 2.5	.115
‐M (SD)	5.6 (3.3)	5.5 (3.3)	6.2 (3.5)	*t* = −1.8	.079
‐Worse/no change/better, *n* (%)	263 (51)/210 (40)/47 (9)	216 (49)/187(42)/42 (9)	47 (63)/23 (30)/5 (7)	*χ* ^2 ^= 5.1	.077
Substance use^c^					
Alcohol	*n* = 328	*n* = 288	*n* = 40		
More/no change/less, *n* (%)	110 (33)/147 (45)/71 (22)	99 (34)/126 (44)/63 (22)	11 (28)/21 (52)/8 (20)	*χ* ^2 ^= 1.2	.567
Illicit drugs	*n* = 62	*n* = 51	*n* = 11		
More/no change/less, *n* (%)	24 (39)/25 (40)/13 (21)	20 (39)/19 (37)/12 (24)	4 (36)/6 (55)/1 (9)		.527
Medication use^c^					
Antipsychotics	*n* = 222	*n* = 185	*n* = 37		
More/no change/less, *n* (%)	54 (24)/144 (65)/24 (11)	47 (25)/117 (63)/21 (11)	7 (19)/27 (73)/3 (8)		.602
Mood stabilizers	*n* = 306	*n* = 293	*n* = 13		
More/no change/less, *n* (%)	46 (15)/241 (79)/19 (6)	44 (15)/230 (79)/19 (6)	2 (15)/11 (85)/0 (0)		1.00
Antidepressants	*n* = 173	*n* = 152	*n* = 21		
More/no change/less, *n* (%)	27 (16)/133 (77)/13 (7)	22 (14)/118 (78)/12 (8)	5 (24)/15 (71)/1 (5)		.579
Anxiolytics	*n* = 178	*n* = 146	*n* = 32		
More/no change/less, *n* (%)	92 (52)/74 (41)/12 (7)	71 (49)/65 (44)/10 (7)	21 (66)/9 (28)/2 (6)		.203
Sleep					
Insomnia symptoms^d^					
‐Number of nights					
Mdn (min−max)	7 (0–14)	7 (0–14)	7 (0–14)	*U* = 15032.0	.166
‐More/no change/less, *n* (%)	212 (41)/272 (52)/36 (7)	181 (41)/236 (53)/28 (6)	31 (41)/36 (48)/8 (11)	*χ* ^2^ = 2.1	.351
Troubling nightmares^d^					
‐Number of nights					
Mdn (min−max)	2 (0–14)	2 (0–14)	3 (0–14)	*U* = 13728.5	*.012*
‐More/no change/less, *n* (%)	133 (25)/362 (70)/25 (5)	108 (24)/316 (71)/21 (5)	25 (33)/46 (61)/4 (5)		.209
Sleep duration before COVID‐19 outbreak h, M (SD)	8.74 (1.8)	8.7 (1.7)	9.3 (2.4)	*t* = −2.8	*.032*
Sleep duration after COVID‐19 outbreak h, M (SD)	8.8 (2.2)	8.8 (1–21)	9.2 (2.7)	*t* = −1.4	.259

*Note*: Bold numerals indicate statistically significant difference between participants with psychotic‐ and bipolar disorders. Due to Bonferroni correction, the significance level was changed: (.05/8) = .006. Italic numerals indicate no longer statistically different after Bonferroni correction.

*Abbreviations*: M, mean; SD, standard deviation; Mdn, median; h, hour.

^a^ Before the COVID‐19 outbreak = before March 12, 2020; after the COVID‐19 outbreak = June 5–July 5, 2020.

^b^Test statistics: Chi‐square, Fischer's exact test, *t*‐test, Mann–Whitney *U*‐test.

^c^Participants who had not experienced these phenomena or did not use substances or medications were excluded from these analyses.

^d^Past 2 weeks.

#### 3.3.3 Social life

Table 4 shows that the majority of participants experienced worsened social life post outbreak, feeling more isolated, outside the community and lonely. Family conflicts had worsened for some.

#### 3.3.4 Substance use

Users of alcohol and illicit drugs reported increased use post outbreak (Table 4).

#### 3.3.5 Medication use

Some participants taking antipsychotics used more post outbreak, with comparatively fewer reporting increased use of mood stabilizers and antidepressants (Table 4). Half of the participants using anxiolytics reported increased use post outbreak.

#### 3.3.6 Sleep

Table 4 also shows data on sleep. Participants reported insomnia symptoms half of the nights and for many this had increased post outbreak. Troubling nightmares were less frequent, but one‐in‐four reported an increase. There were no pre–post changes in sleep duration (*p* = .212).

#### 3.3.7 Mental health services

Table 5 shows that in the total sample and among BP participants significantly fewer participants were in treatment for their mental disorder after compared to before the outbreak, with fewer receiving treatment from general practitioners.

**TABLE 5:** Change in mental health treatment from before to after the COVID‐19 outbreak^a^ in the total sample and across diagnostic groups

	Total sample, *N* = 520			Bipolar disorders, *n* = 445			Psychotic disorders, *n* = 75		
Mental health services before and after the COVID‐19 outbreak,^b^ *n* (%)	Before	After	*p*	Before	After	*p*	Before	After	*p*
‐No treatment	120 (23)	150 (29)	**.001**	98 (22)	129 (29)	**<.001**	22 (29)	21 (28)	1.000
‐General practitioner	194 (37)	167 (32)	**.003**	177 (40)	150 (34)	**.002**	17 (23)	17 (23)	1.000
‐Outpatient clinic	162 (31)	150 (29)	.182	136 (31)	121 (27)	.068	26 (35)	29 (39)	1.000
‐Ambulatory team	21 (4)	22 (4)	1.000	15 (3)	16 (4)	1.000	6 (8)	6 (8)	.508
‐Inpatient treatment	28 (5)	14 (3)	*.011*	13 (3)	5 (1)	.077	15 (20)	9 (12)	1.000
‐Community health worker	95 (18)	79 (15)	*.015*	77 (17)	62 (14)	*.012*	18 (24)	17 (23)	.109
‐Private practice	79 (15)	70 (14)	.064	74 (17)	66 (15)	.077	5 (7)	4 (5)	1.000
‐Other	45 (9)	43 (8)	.855	35 (8)	33 (7)	.839	10 (13)	10 (13)	1.000

*Note*: Bold numerals indicate = statistically significant difference after compared to before the COVID‐19 outbreak. Due to Bonferroni correction, the significance level was changed: (.05/8) = .006. Italic numerals: no longer statistically different after Bonferroni correction.

^a^ Before the COVID‐19 outbreak = before March 12, 2020; after the COVID‐19 outbreak = June 5–July 5, 2020.

^b^Multiple responses could be selected; hence, the percentages do not add up to 100%. Changes from before to after the COVID‐19 outbreak were analyzed with McNemar's test.

Regarding community and charity mental health support, n = 86 participants had support pre‐outbreak. The group difference (PsD *n* = 21 [28%] vs. BD *n* = 65 [15%]; *χ* ^2^ = 7.4, *p* = .007) did not survive Bonferroni correction (*p* = .05/8 = .006). There was reduced support post outbreak for the whole sample; 43 participants (50%) experienced that support was canceled or paused, 22 participants (25%) experienced less support, 17 participants (20%) experienced no change, while 4 participants (5%) experienced more support. There were no diagnostic group differences in change (*χ* ^2^ = 1.5, *p* = .685).

A relatively large proportion of the sample (*n* = 201, 39%) responded that they had not received sufficient treatment for their psychotic or bipolar disorder during the pandemic, and the same proportion of participants (*n* = 200, 39%) reported having received sufficient treatment, while 119 participants (23%) were uncertain. There were no group differences (*χ* ^2^ = 3.4, *p* = .182). Of the *n* = 326 participants who had been in treatment both pre and post outbreak, many (*n* = 140, 43%) had experienced poorer treatment quality post outbreak, 156 participants (48%) reported no change in treatment quality, 30 participants (9%) had experienced improved treatment quality. There were no group differences (*p* = .124).

Of the sample, *n* = 220 participants (42%) had not contacted mental health services post outbreak for issues they normally would, and only 39 participants (8%) had called helplines. There were no diagnostic group differences (*χ* ^2 ^= 2.9, *p* = .087, and *χ* ^2^ = 3.4, *p* = .066, respectively).

Among the *n* = 370 participants receiving treatment post outbreak, 199 participants (54%) had treatment via telephone, and 37% (*n* = 74) wanted to continue this; 86 participants (23%) had videoconference treatment, and 44% (*n* = 38) wanted to continue this; 70 participants (19%) had treatment via text messages or chat, and 40% (*n* = 28) wanted to continue this. A significantly higher proportion of participants with PsD (*n* = 16, 30%) compared to BD (*n* = 54, 17%) had received treatment via text messages or chat post outbreak (*χ* ^2^ = 4.0, *p* = .047), but this difference was no longer significant after Bonferroni correction (0.05/8: *p* = .006).

### 3.4 Impact of pandemic‐related factors on wellbeing and mental health difficulties

Table 6 presents results from the binary logistic regression analyses with *poor wellbeing* as dependent variable and age, being single, worry about pandemic consequences, low coping, loneliness, insufficient treatment, poorer economy, increased alcohol use, and insomnia symptoms entered as independent variables. Insufficient treatment, more loneliness, and low coping significantly and independently predicted poor wellbeing in both the first and the final model.

**TABLE 6:** Logistic regression analyses with poor wellbeing^a^ (no/yes, *n* = 233/287) and poor mental health^b^ (no/yes, *n* = 178/342) as dependent variables

	B	S.E	Wald	*df*	*p*	Ex (B)	95% CI
Predictors of poor wellbeing^c^							
Low coping with the situation (no/yes)	1.558	.213	53.695	1	**<.001**	4.749	3.131–7.205
Loneliness^d^	.197	.034	33.358	1	**<.001**	1.218	1.139–1.302
Insufficient treatment (no or uncertain/yes)	.853	.220	15.016	1	**.000**	2.347	1.524–3.613
Predictors of poor mental health^e^							
Worry about pandemic consequences (no/yes)	.656	.234	7.847	1	**.005**	1.927	1.218–3.049
Low coping with the situation (no/yes)	1.465	.232	40.039	1	**<.001**	4.329	2.749–6.815
Loneliness^d^	.168	.037	20.960	1	**<.001**	1.183	1.101–1.272
Insufficient treatment (no/uncertain or yes)	.795	.232	11.777	1	**.001**	2.215	1.406–3.488
Increased alcohol use (no/yes)	.581	.293	3.929	1	**.047**	1.787	1.007–3.174
Nights with insomnia symptoms past 2 weeks	.054	.024	4.892	1	**.027**	1.055	1.006–1.106

^a^ Poor wellbeing was defined as a score of ≤5 (on a 0–10 scale) and in addition responding that the item had become “worse” after the COVID‐19 outbreak (i.e., after March 12, 2020) on either one of the following items: life satisfaction, meaning in life, social support, or feeling happy.

^b^ Poor mental health was defined as a score of ≥5 (on a 0–10 scale) and in addition responding that the item had become “worse” after the COVID‐19 outbreak (i.e., after March 12, 2020) on either one of the following items: anxious, worried, depressed mood, little interest/pleasure, self‐harm/suicidal ideation, ideas of persecution, or hallucinations.

^c^ Model chi‐square = 171.884 *df* = 3, *p* = .000. The model as a whole explained between 28.1% (Cox and Snell *R* ^2^) and 37.7% (Nagelkerke *R* ^2^) of the variance and correctly identified 75.0% of the cases.

^d^ Loneliness past 2 weeks was scored on a scale from 0 (“not at all”)–10 (“to a great extent”).

^e^ Model chi‐square = 180.081, *df* = 6, *p* = .000. The model as a whole explained between 29.3% (Cox & Snell *R* ^2^) and 40.5% (Nagelkerke *R* ^2^) of the variance and correctly identified 78.5% of the cases.

Table 6 also presents results from the binary logistic regression analyses with *poor mental health* as dependent variable and age, worry about pandemic consequences, low coping, loneliness, insufficient treatment, poorer economy, increased alcohol use, and insomnia symptoms entered as independent variables. Insufficient treatment, more loneliness, low coping, worry about pandemic consequences, more nights with insomnia symptoms, and increased alcohol use significantly and independently predicted poor mental health in the final model. All variables apart from age, poor economy and increased alcohol use were significant in the first model. Increased alcohol use became significant in the model when age and personal economy were removed.

## 4 DISCUSSION

The main findings in this study are that the majority of participants with BD and particularly PsD experienced low levels of wellbeing *and* high levels of mental health difficulties in the early phase of the COVID‐19 pandemic, with around half reporting that they had experienced worsening post outbreak. Participants’ experience of being in recovery from mental health difficulties was significantly lower after compared to before the outbreak. Amongst pandemic‐related factors, low coping with the situation, loneliness, and insufficient treatment had a negative impact on both wellbeing and mental health difficulties, while worrying about pandemic consequences, increased alcohol use, and insomnia symptoms only affected mental health adversely.

### 4.1 Wellbeing and mental health difficulties

Deteriorated wellbeing involved reduced life satisfaction, meaning in life, and positive feelings. Few participants had experienced improved wellbeing. The average wellbeing scores in our participants were approximately 5 and *had worsened* for many, while average scores for life satisfaction and meaning in life in the general population in Norway using the same scale at the same time was over 7 and *had not worsened* (Norwegian Institute of Public Health [NIPH], 2020). Results from the few existing studies on wellbeing and positive mental health in people with PsD and BD are inconsistent in terms of wellbeing being equal to or lower than in healthy controls (Mankiewicz et al., 2013; Stanga et al., 2019; Uzenoff et al., 2010). Our findings suggest that the pandemic affected wellbeing in our target group more adversely than the general population.

Participants reported worsening of especially depression and anxiety symptoms, but also self‐harm/suicidal ideation. This is in line with another survey of people with lived experience of mental health difficulties conducted earlier in the COVID‐19 pandemic finding heightened anxiety and general concerns about becoming mentally unwell because of pandemic pressure (Academy of Medical Sciences, 2020). Others have reported that depression and anxiety symptoms are prevalent reactions to the COVID‐19 pandemic in the general population (Rajkumar, 2020b; Salari et al., 2020), and even higher levels of depression and anxiety in people with PsD and BD compared to healthy controls (González‐Blanco et al., 2020; Van Rheenen et al., 2020). The worsening of self‐harm/suicidal ideation in 33% of participants is highly concerning, as pandemic disruption to mental health services may reduce prevention of suicides (Sher, 2020). Whether our results would be similar in people without a pre‐existing mental disorder is unknown, but a recent study finds no increase in self‐reported mental disorders or suicidal ideation from before the pandemic compared to the early phase of the pandemic (March 12 to May 31, 2020) in the general Norwegian population (Knudsen et al., 2021). Furthermore, this study presents data from the Norwegian Cause of Death Registry, which showed no increase in suicide deaths from March to May 2014–2018 compared to March to May 2020. Also worth noting are findings that levels of anxiety in people seeking help for anxiety and depression increased in the first 4 weeks of the pandemic, but then declined the subsequent weeks (Staples et al., 2020). Whether our target group experienced the same fluctuations in symptom levels is unknown.

In line with expected seasonal increase in mania during spring and summer (Wang & Chen, 2013), 20–50% of the total sample reported increased symptoms of mania; however, only 6% experienced an increase in ideas of grandiosity. As approximately 50% in both groups experienced more irritable mood post outbreak, this item probably captured irritability beyond mania. A prospective study of people with BD found no significant increase of depressive symptoms, but an increase in symptoms of (hypo)mania in the first wave of the pandemic, with a decrease thereafter (Koenders et al., 2021). This suggests that symptoms may fluctuate in line with pandemic changes. Bearing in mind the potential of new mutations of the COVID‐19 virus and new lockdowns, it seems important to increase knowledge of wellbeing and mental health difficulties in different phases of the pandemic.

PsD participants had poorer wellbeing and more mental health difficulties than BD participants post outbreak, but they experienced significantly more worsening than BD participants only in psychotic symptoms. PsD participants also had significantly lower experience of recovery from mental health difficulties post outbreak. Deterioration in hallucinations and ideas of persecution in 20% of participants with PsD is in line with one study (Muruganandam et al., 2020), yet inconsistent with another (Pinkham et al., 2020).

### 4.2 Pandemic‐related factors and their impact on wellbeing and mental health difficulties

The participants were obviously concerned, with high levels of worrying about pandemic consequences, keeping updated, and following recommendations. Worrying about consequences predicted poor mental health, in line with the general population where such worries have been linked to anxiety (Academy of Medical Sciences, 2020). Despite most participants being concerned, as many as 45% reported coping with the situation “a lot.” Nevertheless, *low* coping with the situation predicted both poor wellbeing and poor mental health, suggesting that coping strategies are essential. Almost all participants reported keeping updated via relevant channels, indicating that people with severe mental disorders do keep in touch with world events, contrary to accounts of this population being inadequately informed about the pandemic (Hölzle et al., 2020; Muruganandam et al., 2020). Almost all participants reported that they were following recommendations about hand washing and social distancing, in line with a current study where only 13% of people with severe mental disorders were observed to have problems following protective measures (Mork et al., in press). Studies from previous pandemics of people with PsD found markedly inadequate adherence to protective measures (Brown et al., 2020). The inconsistencies between studies concerning keeping updated and following recommendations may reflect different methodology and different epidemics/pandemics. However, it may also reflect that people with severe mental disorders are heterogeneous, also in the face of a pandemic, and that they require different interventions.

The majority experienced deterioration in their social life post outbreak, presumably in line with the general population. Several COVID‐19 campaigns have been designed around “we‐are‐all‐in‐this‐together” (Nilsen & Skarpenes, 2020; Society of Editors, 2020). This message did clearly not have the full‐intended effect on the majority of our sample, who were feeling *more* outside of the community. The worsening of social isolation and loneliness is not in keeping with the general population, where levels of loneliness remained stable or even decreased during early months of the pandemic (Luchetti et al., 2020; NIPH, 2020). Our findings are in line with previous concerns that social restrictions may impact more severely on people with severe mental disorders (Brown et al., 2020). We found that loneliness predicted both poor wellbeing and poor mental health, consistent with previous findings (Beutel et al., 2017; Eglit et al., 2018). However, over half of the participants experienced social support, with few experiencing a reduction post outbreak. This can be regarded as a resource for interventions aimed to counteract pandemic consequences.

Users of illicit drugs and alcohol reported increased use post outbreak, in line with other studies (Pinkham et al., 2020; Van Rheenen et al., 2020). Increased substance use may reflect maladaptive coping with pandemic distress, anxiety and depression, and/or result from a more monotone/boring life or fewer regulating social constraints. Increased alcohol consumption requires attention, as increased alcohol use predicted poor mental health, as anticipated (Rajkumar, 2020a).

The most prominent finding concerning medication use is that half the participants using anxiolytics reported increased use, probably reflecting pandemic concerns and increase in anxiety symptoms. Whether the increased use of medications was according to prescription or self‐medication is unknown. However, reduced access to general practitioners and mental health services and a reluctance to seek help during the lockdown may have caused participants to self‐medicate.

Insomnia symptoms were prevalent, and for many participants sleep problems had worsened post outbreak. Other studies report more post outbreak sleep disruptions in people with severe mental disorders compared to healthy controls (Solé et al., 2020; Van Rheenen et al., 2020). The worsening is concerning due to adverse outcomes associated with poor sleep (Laskemoen et al., 2019). In line with this, we found that high levels of insomnia symptoms predicted poor mental health.

Significantly fewer BD participants received treatment post outbreak, suggesting that they were not prioritized and/or more reluctant to seek help. In fact, almost half of the total sample had refrained from contacting mental health services for issues they normally would. Bearing in mind the poorer mental health of participants with PsD compared to participants with BD, we regard it as positive that participants with PsD did not report reduction in being in treatment. The group of participants in inpatient treatment before and after the outbreak was small, and the reduction in inpatient treatment in the total sample from before to after the outbreak was no longer significant after Bonferroni correction. A small group of participants received treatment from ambulatory teams, and an interesting finding was that ambulatory care was unchanged post outbreak. Ambulatory teams in Norway provide care for people with *severe* mental health difficulties, including poor functioning, thus maintaining ambulatory care for this group may have been prioritized after the outbreak. Still, more than one‐third of the total sample reported insufficient treatment for their mental disorders and reduced treatment quality post outbreak. Our findings that experienced recovery from mental health difficulties was halved, and that insufficient treatment predicted both poor wellbeing and poor mental health suggests that participants needed the same or *more* treatment in these troubled times. In line with this, studies have found increased mental help seeking and service demand in the early weeks of the pandemic, and later when COVID‐19 transmission was high (Staples et al., 2021; Titov et al., 2020). However, a WHO survey found that 93% of responding countries reported pandemic disruptions to mental health services, despite goals to ensure continue of care (WHO, 2020a), indicating how uncertainty about timing and impact of a pandemic poses challenges for planners and service providers. The Norwegian Directorate of Health (2021) reports that number of adult patients treated in public mental health outpatient clinics remained the same in 2019 and 2020, mainly due to a 50% increase in treatment delivered via phone or videoconference in 2020, while number of admissions to mental health inpatient wards was reduced by 7% from 2019 to 2020. Of note is that these numbers do not reflect number of treatment *sessions* and may not reflect mental health services in the early phase of the pandemic, when they were trying to adjust to the situation. Some of our participants had teletherapy, most prevalently via phone or video conference. For some participants this was an agreeable solution, while others did not want to continue with this mode of treatment delivery, indicating that teletherapy on its own is not adequate for everyone. In many countries, the pandemic also led to increased capacity for mental health helplines (WHOa, 2020). However, few participants in our study had called mental helplines. People with PsD and BD may find helplines to be inadequate to accommodate their specialized needs; continuity in care and an established therapeutic alliance may be especially important for this population.

### 4.3 Clinical implications

The clinical implications of our findings include that low wellbeing and increased anxiety and depression may be normalized as common reactions during a pandemic. New knowledge from this study is that mental health services should be particularly aware of potential increase in self‐harm/suicidal ideation and psychotic symptoms in people with PsD and BD. Moreover, mental health workers should probe actively for signs of loneliness, insomnia, and increased alcohol use and offer adequate treatment. Poor coping should be targeted, for example, by introducing strategies suggested by other people with mental disorders, including cognitive coping strategies (Simblett et al., 2021) and behaviors such as staying connected, keeping busy, physical activity, staying calm, managing media intake, and maintaining routine (Academy of Medical Sciences, 2020). Peer‐support could be beneficial in this context and is currently an underused resource, which should be promoted. Experiences of insufficient treatment suggests that mental health services were not adequately prepared for the COVID‐19 pandemic; however, services should seek to deliver the best *possible* care under current conditions. This population's potential vulnerability and reluctance to seek help implies a need for *active* outreach via available channels. Development of customized teletherapy is needed to fit individual needs. Meanwhile, mental health services should strive to uphold physical sessions when called for, requiring supply and training in infectious control equipment. When conditions prevent optimal mental health services, all available resources should be considered, including family members who already provide important support (Eckardt, 2020). Empowering family members requires establishing contact pre‐crises and supporting them during strenuous times.

### 4.4 Strengths and limitations

Our sample is relatively large, potentially from across the country, with a wide age range. The majority of respondents being female is also seen in other online surveys (Academy of Medical Sciences, 2020; Solé et al., 2020). Diagnosis was based on self‐report; thus, the reliability of the diagnoses and specific diagnostics are uncertain. Moreover, comorbid disorders were not recorded. The sample is skewed towards people with BD. This may at least partly be explained by the user organization for bipolar disorder in Norway sharing the survey to its members, while there is not an equivalent user organization for people with psychotic disorders in Norway. The representativity of our sample may be biased regarding severity of illness and service satisfaction. However, we reached participants with varied levels of wellbeing and mental health difficulties, both in and outside of treatment, indicating a relative diversity. The survey probed experiences pre and post the pandemic outbreak at a single time point, resulting in potential situation and recall‐bias. We lack information about life events not related to the pandemic that may have affected wellbeing and mental health difficulties. However, the survey questions were formulated to encourage pandemic‐related responses. Obvious strengths are that our survey covered both wellbeing and mental health difficulties and a wide range of factors affected by the pandemic. Moreover, people with lived experience of PsD and BD were involved in designing the survey.

## 5 CONCLUSIONS

Our findings indicate that the early stages of the COVID‐19 pandemic had serious consequences for wellbeing and mental health difficulties in many people with PsD and BD. Adverse change in treatment sufficiency, loneliness, insomnia symptoms, alcohol drinking, pandemic worry, and low coping was related to the deterioration. Our findings suggest a need to increase general disaster preparedness in mental health services to ensure provision of sufficient care under suboptimal conditions. Future research should investigate what service users think the health services could do to improve during a pandemic.

### PEER REVIEW

The peer review history for this article is available at https://publons.com/publon/10.1002/brb3.2559

## Data Availability

The data that support the findings of this study are available from the corresponding author upon reasonable request.

## APPENDIX A Survey questions relevant for this paper

### A.1

**Table:**

Questions	Response alternatives
I have:	Bipolar disorder/psychotic disorder
This survey concerns the situation during the corona pandemic, meaning after March 12	
Are you concerned about the consequences of the corona outbreak?	No/yes
Have you followed government recommendations regarding social distancing and hand washing?	Not at all/a little/a lot
**The following questions concern how you are doing now^a^**	
All in all, how satisfied are you with your life now?	0 (not satisfied at all)—10 (very satisfied)
–Has this changed compared to before the corona outbreak?	Become worse/No change/Become better
All in all, to what degree do you experience that what you do in life is meaningful?	0 (not meaningful at all)—10 (very meaningful)
–Has this changed compared to before the corona outbreak?	Become worse/No change/Become better
To what extent do you agree with the following statement: My social relationships are supportive and rewarding? –Has this changed compared to before the corona outbreak?	0 (strongly disagree)—10 (strongly agree) Become worse/No change/Become better
**Think about how you have felt the past 2 weeks. To what extent have you been^a^**	
•Happy	0 (not at all) —10 (to a great extent)
•Engaged •Calm and relaxed	0 (not at all) —10 (to a great extent) 0 (not at all) —10 (to a great extent)
–Have these feelings changed compared to before the corona outbreak?	Become worse/No change/Become better
**To what extent have you experienced the following during the past 2 weeks? (0 = “not at all” and 10 = “to a great extent”)^b^**	
Suicide thoughts or thoughts about self‐harming	0 (not at all)—10 (to a great extent)
–Has this changed compared to before the corona outbreak?	Become worse/No change/become better
Loneliness	0 (not at all)—10 (to a great extent)
–Has this changed compared to before the corona outbreak?	Become worse/No change/become better
A lot more happy/cheerful/elevated than usual several days in a row	0 (not at all)—10 (to a great extent)
–Has this changed compared to before the corona outbreak?	Less/No change/More
A lot more irritable than usual, several days in a row	0 (not at all)—10 (to a great extent)
–Has this changed compared to before the corona outbreak?	Less/No change/More
A lot more active than usual, several days in a row	0 (not at all)—10 (to a great extent)
–Has this changed compared to before the corona outbreak?	Less/no change/more
**To what extent have you experienced the following during the past 2 weeks?**	
Hearing or seeing something that others do not hear or see	0 (not at all)—10 (to a great extent)
–Has this changed compared to before the corona outbreak?	Become worse/No change/Become better
Feeling persecuted or that someone wants to hurt you	0 (not at all)—10 (to a great extent)
–Has this changed compared to before the corona outbreak?	Become worse/No change/Become better
Feeling that you are an extraordinary person or that you have special powers	0 (not at all)—10 (to a great extent)
–Has this changed compared to before the corona outbreak?	Become worse/No change/Become better
Feeling that your surroundings or other people are strange, not genuine or unreal	0 (not at all)—10 (to a great extent)
–Has this changed compared to before the corona outbreak?	Become worse/No change/Become better
Feeling that your thoughts are chaotic	0 (not at all)—10 (to a great extent)
–Has this changed compared to before the corona outbreak?	Become worse/No change/Become better
Feeling that what is communicated on television, radio, internet, or in newspapers is about you in particular	0 (not at all) —10 (to a great extent)
–Has this changed compared to before the corona outbreak?	Become worse/No change/Become better
**Recovery**	
Did you experience that your mental health difficulties were in recovery before the corona outbreak?	0 (not at all) —10 (to a great extent)
Do you experience that your mental health difficulties are in recovery now?	0 (not at all) —10 (to a great extent)
**The following are questions about your life now compared to before the corona outbreak**	
**Treatment**	
Where did you receive treatment for your psychotic disorder or bipolar disorder when the corona outbreak started on March 12? Select all that apply.	No treatment/general practitioner/outpatient clinic/ambulatory (e.g., FACT/ACT team), meeting at home or outdoors)/inpatient ward/community health worker/psychologist or psychiatrist in private practice/other
Where do you receive treatment for your psychotic disorder or bipolar disorder now? Select all that apply.	No treatment/general practitioner/outpatient clinic/ambulatory (e.g., FACT/ACT team), meeting at home or outdoors)/inpatient ward/community health worker/psychologist or psychiatrist in private practice/other
How was treatment delivered when the corona outbreak started on March 12? Select all that apply.^c^	At outpatient clinic or in hospital/via phone/via text messages/via chat/via videoconference (e.g., Skype, Confrere, etc.)/outdoors/at home/other
How is treatment delivered now? Select all that apply.^d^	At outpatient clinic or in hospital/via phone/via text messages/via chat/via videoconference (e.g., Skype, Confrere, etc.)/outdoors/at home/other
Are there any new elements of treatment delivery you would like to continue when the corona pandemic is over? Select all that apply.^d^	Treatment via phone/via text messages/via chat//via videoconference (e.g., Skype, confrere, etc.)/outdoors/other/no
Have you received sufficient treatment during the corona outbreak?	No/yes/uncertain
Has the quality of treatment now changed compared to before the corona outbreak?^d^	Not applicable/worse/no change/better
During the corona outbreak, did you refrain from contacting mental health services with issues you normally would have sought help for?	No/yes
Did you call help lines during the corona outbreak (e.g., Red Cross, etc.)?	No/yes
Did you receive support from community services or charity organizations (e.g., support worker, Fountain house etc.) before the corona outbreak?	No/yes
Did this support change after the corona outbreak?^e^	Paused/canceled/less/no change/more
**Sleep**	
During the past 2 weeks, how many nights did you experience sleep problems (e.g., problems falling asleep, waking up during the night, or waking up too early)?	0–14 nights
–Has this changed compared to before the corona outbreak?	Less/no change/more
During the past 2 weeks, how many nights did you experience troubling nightmares?	0–14 nights
–Has this changed compared to before the corona outbreak?	Less/no change/more
**Before the corona outbreak**:	
Before the corona outbreak: When did you usually go to bed on weekdays?	(Time chosen on a 24‐h clock)
Before the corona outbreak: When did you usually get up on weekdays?	(Time chosen on a 24‐h clock)
**During the corona outbreak**	
During the corona outbreak: When have you usually gone to bed on weekdays?	(Time chosen on a 24‐h clock)
During the corona outbreak: When have you usually gotten up on weekdays?	(Time chosen on a 24‐h clock)
**Use of substances**	
Did your use of substances change compared to before the corona outbreak? –Alcohol –Illicit substances (e.g., cannabis, heroin, and amphetamine)	No use/less use/no change/more use No use/less use/no change/more use
**Medications**	
Did your use of medications change compared to before the corona outbreak? –Antipsychotics (e.g., Zyprexa, Leponex, Risperdal, Ablilify) –Mood stabilizers (e.g., Orfiril, Lamictal, Lithium) –Antidepressants (e.g., Cipralex, Remeron) –Anxiolytics (e.g., Sobril, Valium)	No use/less use/no change/more use No use/less use/no change/more use No use/less use/no change/more use No use/less use/no change/more use
**Social life**	
Has the corona outbreak affected your social life?	Become better/no change/become worse
Did you experience any of the following and has it changed compared to before the corona outbreak? –Feeling isolated –Feeling outside of the community –Family conflicts	Not applicable/less than before/no change/more than before Not applicable/less than before/no change/more than before Not applicable/less than before/no change/more than before
**Keeping updated**	
Which news channels do you use to keep updated about the corona outbreak? Public authorities Newspapers/online newspapers Television/radio Social media Blogs Friends/family	Not at all/a little/a lot Not at all/a little/a lot Not at all/a little/a lot Not at all/a little/a lot Not at all/a little/a lot Not at all/a little/a lot
**Coping**	
Do you experience that you are coping with the situation during the corona outbreak? **Personal economy** How is your economy after the corona outbreak?	Not at all/a little/a lot A lot worse/a little worse/no change/a little better/a lot better

^a^ The questions about life satisfaction, meaning in life, social support, and positive feelings were taken from the Norwegian guideline list for measurement of wellbeing/quality of life (Nes et al., 2018), which is based on the OECD Guidelines on Measuring Subjective Well‐being (OECD, 2013) and Diener's Flourishing Scale (Diener et al., 2009).

^b^ Questions about symptoms of depression and anxiety from the Patient Health Questionnaire‐4 (Kroenke et al., 2009) were included in this section.

^c^ This question was only given to participants who had responded that they were in treatment on March 12.

^d^ This question was only given to participants who had responded that they were in treatment at the time of the survey.

^e^ This question was only given to participants who had responded that they had received support from community or charity organizations before the COVID‐19 outbreak.
